# Catarrhal Conjunctivitis—“Sore Eyes”

**Published:** 1883-06

**Authors:** Chas. W. Hickman

**Affiliations:** Augusta, Ga.; Lecturer on Diseases of the Eye in the Medical Department of the University of Georgia


ATLANTA

### MEDICAL REGISTER.

VOL. II.] JUNE, 1883. [No. 9

### Original.

[*Written for the Transactions of the Medical Association of Georgia*, 1882]

### CATARRHAL CONJUNCTIVITIS — "SORE EYES."

BY CHAS. W. HICKMAN, M.D., OF AUGUSTA, GA.
Lecturer on Diseases of the Eye in the Medical Department of the University of Georgia.

Catarrhal conjunctivitis is an inflammation of the conjunctival mucous membrane, both that portion lining the lids as well as that covering the exposed portion of the ball. It is characterized by a considerable amount of redness and swelling, puffiness of the lids with a yellowish discharge coming from between them ; has a certain course to run, and although alarming at times in its features, presents a tendency always to get well if not injudiciously treated by strong caustic and astringent lotions. This inflammation occurs most frequently in children, and in many respects closely resembles the exanthemata as measles, scarlet fever and the like, being quite contagious, often occurring but once in a lifetime, and as before intimated, has its own course to run. We see it at times sweeping a section of country as a frightful epidemic known commonly under the name of "sore eyes." In our own section such an epidemic may be looked for almost annually about harvest time. Its contagious powers are not so great to grown persons, and, indeed, they often present a perfect immunity from it; but it is surprising to see how much so it is to children; if a child is brought within twenty feet of another suffering from it, he is almost sure to fall a victim. We can easily see, then, that where one child in a house takes it, the others should be immediately isolated. The swelling sometimes attains to a considerable degree, the lids are puffy and thick, and if from being forcibly pulled apart from their matted together condition, a thick yellowish discharge presents itself, and sometimes, indeed, almost spurts out into your face. That portion of the membrane covering the eye-ball may be so swollen and œdematous as to form quite a mound all around the clear margin of the cornea, making the condition known as chemosis. One of the most frequent symptoms in the early stages is a feeling as if sand or grit had become lodged under the lids, and is due to the friction of the swollen papillæ over the eye-ball as the lids move back and forth during winking. This important symptom should always be borne in mind, for it not unfrequently happens that patients consult us at the commencement of the disease, for the removal of a foreign body, and can scarcely be made to believe that none such is present, even after a most careful and scrutinizing inspection. Although statements are made to us that the affection is not attended with much pain, yet in well marked cases such as I have just been describing, this is far from true, for we find that during the height of the attack the patient suffers from considerable pain and restlessness, which markedly increase towards night, paroxysms coming on at times which are unendurable, except under the use of opiates.

Catarrhal ophthalmia may arise from any of the causes which may give rise to a simple hyperæmia of the conjunctiva, such as cold, wind, glare, over-use and the like, but in my own opinion most frequently arises entirely independent of all this, just as one of the exanthemata, and largely propagated by contagion. Of all the affections lightly passed over both by physicians, as well as those outside of the profession, it may be pre-eminently mentioned. Nothing is more common than to see a whole family, especially the children, all affected with sore eyes, and the matter treated as of no importance. The oculist, however, has long known that it carries with it more weight than is usual to ascribe to it, and unless unpleasant complications are early recognized and dealt with, unhappy results are but too sure to follow. Ulcers of the cornea are by no means of such unfrequent occurrence as some authors would lead us to suppose, and I need scarcely mention the baneful effect of the use of strong astringent or caustic lotions in such instances. It is far from improbable that ulcers are even produced at times by their use. The cornea should at all times and under all circumstances be carefully watched, but especially in children of delicate health or who have inherited a scrofulous or specific taint. Very little is to be feared from the inflammation of the mucous membrane itself so long as the cornea remains intact. Should any haziness or steaminess, or any distinct ulceration make its appearance, the mucous membrane may be entirely overlooked for the time being, and all our efforts directed towards the preservation of the more delicate corneal structure. Fortunately, if a few simple principles are but borne in mind, we may almost always succeed in accomplishing this object. One or two interesting cases may well illustrate this point.

Mrs. S., while in delicate health, her system much run down from the prolonged effects of malarial fever, contracted a severe case of "sore eyes" Under the injudicious advice of a friend, she made use of an eye water, considered a specific, and which on investigation proved to be a solution of nitrate of silver. For a long time her sufferings were very great, and finally she was induced to try if special aid could not do something for her relief. On examination it was found that a large central ulcer had perforated its way through the cornea and attended with prolapse of the iris, and extensive synechia. Had careful bandaging been resorted to, atropia freely instilled into the eye, and this combined with the internal administration of quinine and tonics, I am quite sure that a good result would have been obtained.

Mrs. M. and daughter were both the victims of an attack of "sore eyes." Here, again, a well-meaning friend advised the use of an eye water having great specific powers, and which on subsequent investigation proved to be a strong solution of nitrate of silver. At the time of consultation, in the case of the mother, a large sloughing ulcer at the upper edge of the cornea had already produced effects which nothing could remedy. The child had only used the eye water a few times, but a central ulcer of large size was the immediate result. In both cases the other eye was not affected until two or three days after consultation, and with bandaging, cleanliness and atropia, combined with tonics and quinine internally, the inflammation ran its course to a successful termination. Even in the child, by quickly stopping the use of the nitrate of silver, the ulcer already formed readily yielded, leaving only a slight cicatrice as a result.

In the treatment of catarrhal conjunctivitis, our first care should be scrupulous cleanliness. The eye should be bathed several times a day with tepid water (one drachm of chloride of soda to the pint of tepid water) care being taken that all the matter is removed. After the bathing, a light compress bandage should be applied, for by thus keeping the eyes at rest, great relief is afforded. This alone is often sufficient to effect an easy yet complete cure. For a long while I was greatly perplexed to find some means of controlling the intense ciliary neuralgia so unendurable at times, especially at night. In grown persons the hypodermic injection of the fourth of a grain of morphine is undoubtedly the quickest relief, but as children, and especially very young children, do not stand opiates well, some other means should be resorted to. I find that the extract of hyosciamus, when we can rely upon its uniformity of strength, answers the purpose most admirably. By getting the patient well under its influence by bed-time, a good night is almost sure to follow. That prepared by Edward Squibb, of Brooklyn, I find to be always uniform, and prefer it to all others. My method of using it is to combine it with sulphate of quininæ, giving $\frac{1}{12}$ gr. to $\frac{1}{2}$ gr. of sulph. of quininæ every hour before bed-time until four or five or even six doses have been taken, as the child seems able to stand it. This is to be given to a child two years of age, the dose being increased according to the age of the patient as the physician's judgment dictates. I seldom find that adults are able to take more than two or three doses 1 gr. each of Squibb's preparation without showing decided symptoms of mild hyosciamus poisoning, and as we do not wish to bring about this result, the administration of the drug should be with some degree of care. Should these symptoms be unpleasant, a hypodermic injection of morphine is the most speedy relief. I can speak with great confidence from a very large experience of this method of dealing with the ciliary neuralgia, and can state that in my own hands it has proved almost a specific. Furthermore, the unpleasant effects which naturally follow the use of opiates, are almost entirely done away with in this method of treatment, the patient awakening the next morning refreshed and invigorated.

I am by no means partial to the use of astringents in the acute stages of the disease, but when these have passed, I think, provided the cornea is clear, they assist us in hastening the termination of the inflammation. Mr. Critchett, of London, has well remarked that we are too apt to load up our gun with zinc and fire aloose, and the untold devastation produced we could scarcely believe. Astringents should never be too strong and never under any circumstances should we make use of lead, first because we have a large variety of others equally as good, if not better to select from, and secondly, the deposit of it upon the cornea would be anything but pleasant for the physician, and such a deposit is sure to take place should any ulceration of the cornea be present, as the following case will show: Mr. H, a gentleman from Edgefield Co., S. C., consulted me for a large white spot on the sight of his eye. He stated that while suffering from an attack of sore eyes a prescription had been given him by a friend, the fact naturally not being known of the existence of a large central corneal ulcer. The ulcer fortunately healed without bursting, but deep in its bed was a thick deposit of lead which all known methods could not effectually remove.

I have even seen the lead deposits show themselves where the abraided epithelial portions of the cornea were too small to be seen by the eye alone.

Nitrate of silver, I think, only needs further mentioning to be condemned.

By following out the few broad principles which experience teaches, should guide us in the treatment of this affection, and looking upon it more in the light of a corneal than a conjunctival trouble, we have nothing to fear in regard to an unpleasant termination. Although patients are frequently seen by us, where neglected corneal complications, or their injudicious treatment have produced troubles which could never be remedied, yet, in a very large experience, I have never met with a case which if taken in time could not be successfully treated.

